# Inquiry-Based Learning and Conceptual Change in Balance Beam Understanding

**DOI:** 10.3389/fpsyg.2020.01621

**Published:** 2020-07-22

**Authors:** Joep van der Graaf

**Affiliations:** Behavioural Science Institute, Radboud University, Nijmegen, Netherlands

**Keywords:** conceptual change, scientific reasoning, inquiry-based learning, latent transition analysis, strategy use

## Abstract

Inquiry-based learning has the potential to foster conceptual change, but whether it can induce an advancement in strategy use is not yet known. Such an advancement seems plausible, because conceptual change can be reflected in the use of new strategies. Whether inquiry-based learning leads to advancement in strategy use can be tested with strategy-based tests, such as the balance beam test. Distinct strategies have been proposed and identified for this test. Therefore, the present study compared response patterns on the balance beam test before and after an inquiry-based lesson. The experimental condition completed a digital inquiry-based lesson about the balance beam (*n* = 113), and the control condition completed a similar inquiry-based lesson but investigated a different topic (*n* = 44). The participants were aged 8–13 years old and were unfamiliar with the law of moments. The balance beam test (pretest and posttest) consisted of 25 items. Overall accuracy in solving balance beam problems improved after the inquiry-based lesson in the balance beam (BB) condition but not in the control condition. Classes, identified with latent transition analysis (LTA), appeared to be globally in line with previously identified strategies in the balance beam test. Condition was entered as a covariate in the LTA to identify which changes in strategy use could be attributed to the experimental intervention. First, changes from pretest to posttest were found, which supported that a change in strategy use occurred in some children. Second, there were more improvements in the BB condition, and these improvements indicated larger gains compared to the control condition. This means that in science education, it is important to map out prior knowledge and its effect on learning paths. Overall, results suggested that conceptual change could be measured as a change in strategy use and modeled with LTA to reveal that 26% of the children showed conceptual change after a single inquiry-based lesson.

## Introduction

Inquiry-based learning entails discovering scientific laws through the process of investigation (Dunbar and Klahr, 2012). It is relevant for contemporary education in which both knowledge and skills are taught (Fischer et al., 2014). Inquiry-based learning is useful in this regard, as it promotes the acquisition of scientific reasoning skills and scientific knowledge (Lazonder and Harmsen, 2016). During the development of scientific reasoning skills and construction of scientific knowledge, conceptual change takes place. Conceptual change refers to children’s gradual revision of beliefs from intuitive (and often incorrect) to complete and correct conceptions; these conceptions, either intuitive or complete, are called explanatory frameworks (Vosniadou, 2002). Explanatory frameworks can be measured as strategy use when solving problems, because strategies reflect children’s explanatory frameworks. Previous studies have revealed that, especially, young children (i.e., primary school children) are susceptible to conceptual change (Vosniadou, 2002) and that inquiry-based learning can foster conceptual change (Huang et al., 2017). However, little is known about the effects of inquiry-based learning on strategy use. Therefore, the present study investigated strategy use before and after a digital inquiry-based lesson in primary education. In the present study, conceptual change was measured as a change in strategy use when solving balance beam problems. To foster conceptual change, an inquiry-based lesson about the balance beam was used and compared to a control condition where participants completed an inquiry-based lesson about a different topic, namely, gears.

Conceptual change theories describe how people mentally store and organize information and how this information and its organization change when people learn. There are debates about what a concept is and how change takes place (e.g., Piaget, 1978; Posner et al., 1982; Chi and Roscoe, 2002; diSessa, 2002; Vosniadou, 2002). Concerning the question of what a concept might be, Vosniadou (2002) definition will be used, as it deals with young children’s science learning. Conceptual change assumes that there is an initial mental model, or explanatory framework about scientific phenomena (Vosniadou, 2002). These explanatory frameworks embody causal notions and allow distinct types of explanations and predictions (Carey, 1985; as cited in Vosniadou, 2002). Explanations and predictions are constrained by young children’s conceptual system, which drives the interpretation of their observations and the information they receive.

For example, when considering the law of moments as a scientific phenomenon that explains which side of a balance beam goes down when placing weights on it, then an example of an explanatory framework is the predictions about which side of a balance beam goes down and the explanations why. A balance beam (or seesaw) has two sides on which different amount of weights can be placed at different distances from the center (i.e., fulcrum). The simplest explanation proposed by Siegler (1976) is that only weight on either side of the balance beam determines which sides goes down (Strategy 1), while the most complex (and the only correct) explanation is that weight and distance on either side should be multiplied and compared to determine which side goes down (Strategy 4). In between are Strategy 2, which is like Strategy 1, but distance is also used when weights are equal, and Strategy 3, using both weight and distance but in an inconsistent manner.

Explanatory frameworks such as these guide learners’ behavior in science tasks. Therefore, explanatory frameworks can be measured through a learners’ strategy use, and conceptual change can be seen as a change in strategy use. For conceptual change to occur, children can advance from simple strategies that they need to abandon to be able to adopt new (and potentially more complex) strategies. Note that this assumes that initial strategies are error prone and/or incomplete, which is often the case in primary school children (Vosniadou, 2002).

This brings about the question about how change takes place. Conceptual change takes place in roughly two ways: explanatory frameworks themselves change, or the relations among explanatory frameworks change (see diSessa and Sherin, 1998, for a review). For example, in the first case, distance can be added as a factor to the explanatory framework of the law of moments. In the latter case, how weight and distance are combined to determine which side of a balance beam goes down might change from “add weight and distance on either side and then compare the sides” (Addition Strategy) to “multiply weight and distance on either side and then compare the sides” (Strategy 4). There is evidence from studies on young children’s understanding of force, matter, heat, etc. that these changes occur slowly (Vosniadou, 2002). Chinn and Brewer (1998) further specify how explanatory frameworks transform describing the degree of change from zero change to complete change: children can ignore data, reject data, question data’s validity, exclude data from their respective theory, suspend data (abeyance), reinterpret data, accept data and make peripheral changes to the current theory, or accept data and change theories. To summarize, conceptual change is conceptualized as a change in explanatory frameworks themselves and/or a change in the relations between explanatory frameworks, and both types of changes can range from no change to full change.

The current study explored how inquiry-based learning can promote conceptual change. Although explanatory frameworks might not easily change and conceptual change seems to be a slow process (Vosniadou, 2002), inquiry-based learning has several features that help to promote conceptual change. Inquiry-based learning enables children to gain insight into scientific concepts by engaging in scientific reasoning activities, such as generating hypotheses, conducting experiments, and drawing conclusions (Klahr, 2000). During the first step, generating hypotheses, children think about and explicate what might explain a specific scientific phenomenon, such as how weight might affect which side of the balance beam goes down. In the second step, experimentation, children set up an experiment by selecting settings of variables, such as selecting a weight of 5 kg to be placed on the left side of a balance beam, and observe what happens: left side goes down, right side goes down, or the beam is balanced. The final step, drawing conclusions, is when children interpret what they have observed and explain it, such as that the left side of the beam went down because there was a weight on the left side and no weight on the right side. These scientific reasoning activities should be well guided in order to be effective in enhancing learning (see Lazonder and Harmsen, 2016 for a meta-analysis).

Several processes of inquiry-based learning seem to foster conceptual change. Conceptual change is hampered by children’s lack of orderly organization when explaining and predicting scientific phenomena, bias in evaluating evidence, and problems falsifying or verifying understanding (Vosniadou, 2002). The first problem can be addressed by inquiry-based learning, as children should articulate their explanations and predictions when generating hypotheses, which is one of the first steps in the inquiry cycle. Secondly, self-generated evidence might help to overcome bias in evaluating it (Mussweiler and Strack, 1999), and perceiving evidence can help in creating a new explanation, instead of distorting the evidence (Koerber et al., 2017). Third, inquiry-based learning revolves around proposing explanations and testing them in experiments (Dunbar and Klahr, 2012), which is a process of explicating understanding that subsequently might be falsified or verified. Taken together these features of inquiry-based learning have the potential to induce conceptual change.

There is empirical support that guided inquiry can foster conceptual change. Huang et al. (2017) designed an eight-lesson inquiry-based unit for 13- to 14-year olds that comprised six investigations. During the eight lessons, students were introduced to the topic: motion and force. Each investigation was about a different specific topic, such as changing speed. During investigations, students predicted what the effect of a variable would be (i.e., hypothesis generation), observed what the effect was by designing and conducting experiments, and explained what the effect was (i.e., conclusion). Conceptual change was operationalized as a decrease in misconceptions, an increase in correct propositions, and a correct organization of propositions. Misconceptions were assessed by a multiple-choice test, and the propositions and their organization through a concept map. The results showed that children held fewer misconceptions and more correct propositions after the inquiry-based lesson series—although no improvement was observed in the organization of propositions. Despite this clear advancement of understanding, it remains unclear whether the guided inquiry also led to usage of different explanatory frameworks. A strategy-based assessment of conceptual change is needed.

In addition to evidence about which features of inquiry-based learning might foster conceptual change, there is evidence about how inquiry-based learning might foster conceptual change. In the case of the balance beam task (Siegler and Chen, 1998), through inquiry-based learning, children should notice potential explanatory variables, formulate new explanations, generalize to new problems, and maintain the new strategy to learn new strategies. Inquiry-based learning revolves around these processes. Initial strategies, for example, Strategy 1 (use weight only), can be put to the test. When the results do not match predictions, for example, when the side with the largest weight does not go down, children might notice the effect of distance. Noticing potential explanatory variables seems to be the most important factor in learning new strategies (Siegler and Chen, 1998). The results from these experiments can be used to formulate new predictions. Subsequently, new predictions, such as those in line with Strategy 2 (use distance when weights are equal), can be generalized and tested. The results then might show support for the new predictions. When children explain these findings, they might (partially) adopt and maintain a new strategy, Strategy 2 in this case.

There is large variation within and between children in which strategies are used at a given time and how the strategies that are used change. Therefore, in order to model conceptual change, this natural variation within and between children should be taken into account by appropriate statistical models. Latent models, in particular, are appropriate for two main reasons. Firstly, explanatory frameworks show qualitative and quantitative differences between children, and secondly, explanatory frameworks show variation at specific time points, as well as over time (see Hickendorff et al., 2018, for details). Therefore, statistical models should allow intra- and inter-individual differences at a single time point but also between time points. Models with these features have been applied to strategy-based tests that can measure explanatory frameworks in use. A common example is the balance beam test (Inhelder and Piaget, 1958; Siegler, 1976), which lends itself well to latent class analysis (LCA) (Jansen and van der Maas, 1997). LCA identifies a number of homogeneous subgroups, called classes, in the heterogeneous data of the whole group at a specific point in time and has frequently been used to model strategy use when solving balance beam problems (Jansen and van der Maas, 1997, 2002; Boom et al., 2001; Boom and Ter Laak, 2007). Two common findings are that (1) five or six classes are found and (2) Strategies 1, 2, and 4 are identified as separate classes. Strategy 3 often is not found as such, but a variant can be detected. Instead of complete uncertainty when weight and distance contradict which side goes down (i.e., Strategy 3), children may use the so-called Addition Strategy (Wilkening and Anderson, 1982): weight and distance on both sides are added and then compared (e.g., Boom et al., 2001; Boom and Ter Laak, 2007). The study by Boom et al. (2001) investigated strategy use of 7- to 14-year-old children. In addition to Strategies 1, 2, 4, and the Addition Strategy, two classes (out of six) did not match any strategy. A later study with 5- to 14-year-old participants and two covariates of class membership (age and working memory) revealed comparable classes, but the class representing Strategy 4 was not found (Boom and Ter Laak, 2007). Boom and Ter Laak (2007) attributed the absence of Strategy 4 to the inclusion of only few older participants. These studies show the feasibility of using LCA to identify strategy use and thus capture children’s explanatory frameworks.

Latent transition analysis (LTA) models conceptual change by extending LCA to multiple time points. LTA creates classes, as LCA does, but it also creates transition probabilities that indicate the likelihood of moving from one class, at a point in time, to another, at a later point in time (Bartolucci et al., 2014). LTA has previously been applied to inquiry-based learning settings. Schneider and Hardy (2013) studied the effects of a constructivist intervention about floating and sinking using three conditions: baseline, low instructional support, and high instructional support. Participants were children from 8 to 11 years old. Five profiles were found ranging from misconceptions to scientific conceptions. From pretest to posttest (1 week after the intervention), most children improved their understanding, and from posttest to retention test (1 year later), most children did not change. In addition, more instructional support was related to more transitions, indicating improvement. Another example is a study on change in knowledge structures after 15 lessons, which included inquiry-based learning (Edelsbrunner et al., 2018). The authors analyzed primary school children’s (ages 6–13 years) knowledge about floating and sinking. Medium-sized effects were found that showed a decrease in misconceptions and an increase in correct conceptions. LTA showed that the largest change was a transition from moderate misconceptions to low misconceptions, indicating a restructuring of children’s knowledge. These results are promising in light of studying conceptual change, as LTA was more informative than regular regression-based analyses, which do not take into account differences in students’ knowledge and its development (Edelsbrunner et al., 2018).

To summarize, conceptual change can be observed in a change in strategy use, but this notion has received little attention in empirical research. Furthermore, it is yet unknown what the effect of inquiry-based learning on advancement in strategy use is, while it can be disentangled using informative statistical models (Hickendorff et al., 2018). Therefore, the present study investigated strategy use when solving balance beam problems, before and after a digital inquiry-based lesson with a virtual lab about balance beams (BB condition) compared to a control condition. In both conditions, children completed the balance beam test before and after an inquiry-based lesson, but the control condition studied a different topic, namely, gears. Thus, it was the aim to detect change in strategy use when solving balance beam problems after an inquiry-based lesson about the balance beam, compared to a control group that also completed an inquiry-based lesson but on a different topic. The research questions were: (1) to what extent does inquiry-based learning about balance beams (versus inquiry-based learning about gears) promote advancement in strategy use when solving balance beam problems, and (2) to what extent can change in strategy use be measured with the balance beam test and modeled with LTA? In addition, effects of age and gender on overall accuracy at pretest and posttest, and on the difference score (posttest minus pretest), were explored.

Regarding strategy use, it was hypothesized that five or six classes would be found and that Strategies 1, 2, and 4 and the Addition Strategy would be detected, as well as one or two classes that could not be classified, because that is what previous studies with LCA of balance beam performance showed (Jansen and van der Maas, 1997, 2002; Boom et al., 2001; Boom and Ter Laak, 2007). It was expected that accuracy in the present study would be lower than in the study by Boom et al. (2001) because they used four age groups to estimate the classes and the present study targeted a single, younger age group, 10 years and 9 months on average.

With reference to conceptual change, the inquiry-based lesson about the balance beam was hypothesized to be effective in teaching strategies that are more advanced to children. An improvement in strategy use from pretest to posttest was expected in the BB condition, while no improvement was expected in the control condition. In line with these expectations, class membership was expected to change from pretest to posttest in the BB condition and not in the control condition. To assess change of class, LTA was used. It was hypothesized that children would advance in the BB condition from classes matching a simple strategy to classes matching the next strategy, indicating slow advancement (Vosniadou, 2002).

## Materials and Methods

### Participants

A total of 157 children from grades 4, 5, and 6 participated. The BB condition included 114 children, and the control (gears) condition, 45 children. Age ranged from 8 years and 5 months to 13 years (*M* = 10 years and 9 months, *SD* = 1 year). The children had not learned about the law of moments or gears yet. Children were not familiar with digital inquiry-based learning, but they had experience with digital learning materials and with inquiry-based learning without digital media.

Participants came from five schools and eight classrooms. Schools agreed to participate in the present study and helped to inform the parents and/or caretakers. Two schools chose to ask for consent actively, and three chose the passive approach. Consent was obtained from the parents and/or caretakers. Children and their parents and/or caretakers were given information about the present study and were given the opportunity to ask questions.

### Design

Classrooms were assigned randomly to one of the conditions, while making sure that more classrooms would be assigned to the balancing beam condition to obtain a larger sample size for transitions in the LTA. The procedure was the same in both conditions: There were three sessions; each on a separate day with 1 week between them. In the first session (pretest), the balance beam test was administered (duration was 15 min). The second session consisted of the digital inquiry-based lesson (duration was 45 min). The third session (posttest) consisted of the balance beam test. Children also completed other tests during the first and third sessions, as part of a larger study.

All tests and the digital inquiry-based lesson were performed on a computer. Children went to the computer room, class by class, or laptops were used in the classroom. The instruction was presented digitally. Before children started, the experimenters instructed them to log in and proceed with the instruction and tests. The teacher was present during all sessions to manage the classroom.

### Materials

#### Inquiry-Based Learning Environment

Children in both conditions completed a digital inquiry-based learning lesson. The BB condition studied the effects of weight and distance on the balance of balance beams. The control condition investigated gears, their direction of rotation when creating chains of at least two gears, and acceleration when connecting different sizes of gears. The lessons were similar in all aspects (introduction, increase in difficulty, sequence of activities, and duration) except for the topic of investigation. Thus, both lessons were comparable in rigor and complexity. Children worked at their own pace. There were no practice trials at the beginning, but there was instruction on how to use the digital learning environment. An experimenter was present to answer questions regarding the use of the learning environment.

In the introduction in the BB condition, a problem was presented in which a seesaw did not seem to work because the mother was heavier than the child was. It was suggested that asking a friend to join (i.e., change the weight) or asking the mother to move closer to the fulcrum (i.e., change the distance) might solve the problem, with pictures of the problem and both suggestions. Next, in the experimentation steps, children were instructed to manipulate one variable (weight or distance) and keep the other one constant (distance or weight). This was done first for weight and then for distance. For the effect of both, children were instructed to manipulate both weight and distance. Furthermore, in all experimentation steps, children could type information about the experimental design and the outcomes in an input box. The last part of the instruction in the experimentation phase was that after several tries, they were asked to explain, as they would to someone who did not understand it yet, how they managed to balance the beam and what the effect of weight, distance, or both was on balance.

**FIGURE 1:**
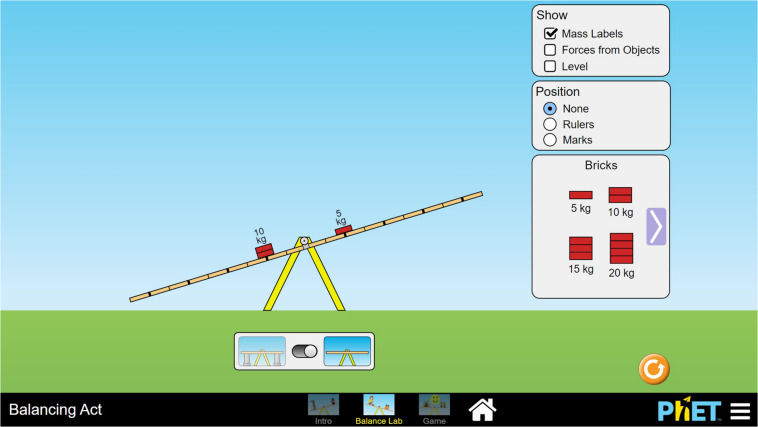
The virtual lab with the balance beam that was used during the inquiry-based lesson.

Experiments were conducted by manipulating weight and distance in a virtual lab; see Figure 1. The lab directly showed the effect of the manipulations on the balance of the beam, which provided feedback to the children about the role of the variable(s) they manipulated. The brief explanation in the final step included the notion that both weight and distance matter for the balance of a beam, and there was a short quiz, so children could use their newly acquired knowledge. The short quiz consisted of two multiple-choice questions with four options about where to place a person on one side of a seesaw to make sure that it would be balanced. There was feedback on the correctness of the response and an explanation about the correct option. A hint was available, and when clicked, it showed an example in which torque was calculated. Finally, children were thanked for their participation.

In the control condition, the introduction presented a problem of a boy who wanted to cycle backward and do it fast. In the experimentation steps, the number of gears and direction of rotation, the type of connection between gears (direct or with a belt) and direction of rotation, and different sizes of gears and acceleration were investigated. The lesson ended with more information about gears, direction of rotation, and acceleration.

#### Balance Beam Test

**FIGURE 2:**
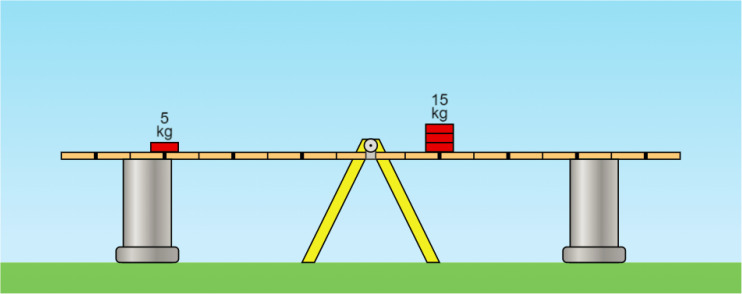
An item of the balance beam test.

The balance beam test aimed to assess strategy use when solving problems with the balance beam (Inhelder and Piaget, 1958; Siegler, 1976). Children were asked to decide which side of the balance beam would go down, left, right, or neither (balance). The weights and distances as described by Boom et al. (2001) were used in the same order to create 25 items divided into five sets, consisting of five items each.^1^ The first set of five items addressed weight. The distances were the same on each side of the beam, but the weights were not. The second set of five items addressed distance: weights were the same, but distances were not. The third set of five items addressed balance: weights and distances were different but in such a way that the beam would balance. The fourth set of five items addressed conflict-weight, which means that the larger weight goes down, but distance on that side is smaller than on the other side. The last set of five items addressed conflict-distance, in which the side with the larger distance does go down, but weight on that side is smaller. The weights and distances in conflict-weight and conflict-distance items were different for each side of the balance beam. A digital version of the test was created for the present study. Screenshots with various setups of a balance beam in a virtual lab, called Balancing Act, were used; see Figure 2. Balancing Act was produced by the PhET project at the University of Colorado, Boulder^2^. At the bottom of the screen, a calculator could be accessed. If children wanted, they could access it to do calculations needed to solve the problems during the test. When using Strategy 1 (weight only), the weight and conflict-weight items should be answered correctly, and the rest incorrectly. Strategy 2 (use weight and, when weights are equal, use distance) should lead to correct responses on weight, conflict-weight, and distance items but incorrect responses on conflict-distance and conflict-balance items. When applying Strategy 3 (using both weight and distance but in an inconsistent manner), weight and distance items should be answered correctly, and all other items should have accuracy around chance (33% correct). The Addition Strategy (add weight and distance) would lead to correct answers on weight, distance, and conflict-balance items, as well as three of the conflict-weight items and one conflict-distance item; see Appendix A. Finally, when all items are answered correctly, accuracy indicates usage of Strategy 4 (use weight and distance correctly). The test consisted of 25 items in total, and for each item, one point could be earned. The test was reliable (Kline, 1993) at pretest, α = 0.72, λ_2_ = 0.77, and posttest, α = 0.73, λ_2_ = 0.77.

### Analyses

The analysis of balance beam performance on pretest and posttest mainly followed the analyses by Boom et al. (2001). The number of test items answered correctly was calculated for each child. One control participant had more than six unanswered items on pretest and was removed from further analyses. All other participants did answer at least 19 items. Unanswered items were recoded as incorrect, because missing per variable was low (maximum 4.5%) and visual inspection revealed no common pattern of missingness.

LTA was used to assign response patterns on all items to latent classes. The classes were based on both pretest and posttest; thus, each participant contributed two sets of responses in the LTA. Classes were categorized and named based on their resemblance to Siegler’s strategies (1976). In addition, transition probabilities were calculated that indicate the likelihood of moving from one class at pretest to another at posttest. Condition was added as a covariate of transition probabilities. To match the analysis by Boom et al. (2001), who used LCA, a latent Markov (LM) model for longitudinal data was used, which has the same features, namely, Bayesian inferencing and Gibbs sampling (Bartolucci et al., 2014). This is also in line with the LTA by Edelsbrunner et al. (2018). The LMest package (Bartolucci et al., 2017) was used to conduct LTA with the present data using LM models. This package is available in R (R Core Team, 2019). Transition probabilities were set to be dependent on time, which means that transitions from pretest to posttest only depended on class assignment at pretest. The tolerance level was set to 10e^–8^. The maximum number of iterations of the algorithm was set to 1,000. This resulted in conditional response probabilities per item for each class, also called item probabilities, which indicate the probability to answer the item correctly. In addition, transition probabilities were calculated for each class on pretest to each class at posttest. Finally, for each participant, the probability to belong to a class was calculated per class. Note that one participant can be assigned to multiple classes, but often, one class is dominant (over 75% assignment). This analysis takes into account effects per time point and over time when calculating class assignment and transition probabilities (Bartolucci et al., 2017).

## Results

### Comparing the Balance Beam and Control Condition

The first set of analyses examined whether participants in the BB and control conditions differed in age, gender, and test performance (pretest, posttest, and difference score); see Table 1. Participants in the control condition were slightly younger than in the BB condition. Other comparisons between the conditions revealed no difference in gender or pretest scores but a higher posttest and difference score (posttest minus pretest) for the BB condition.

**TABLE 1:** Descriptive statistics and comparison of the balance beam (BB) and control conditions on gender, age, pretest and posttest balance beam scores, and the difference between pretest and posttest.

	BB			Control						
	*M*	*SD*	*N*	*M*	*SD*	*N*	*df*^1^	*t*	Adj. *p*	*d*
Gender^2^	1.48	0.50	105	1.41	0.50	44	147	0.75	0.457	0.13
Age*	10.60	1.08	102	11.00	0.69	44	122.61	2.67	0.014	0.41
Pretest	13.90	3.86	113	13.36	4.02	44	155	0.80	0.457	0.14
Posttest*	15.28	3.34	113	12.57	3.78	44	155	4.41	< 0.001	0.78
Difference score*	1.38	3.11	113	–0.80	3.91	44	155	3.52	< 0.001	0.65

^1^When variances were unequal, degrees of freedom of the t-tests were adjusted. ^2^Girl = 1, boy = 2. *p < 0.05.

### Age and Gender Effects on Balance Beam Performance

The second set of analyses examined how age and gender were related to test performance. Correlations were calculated first. Age and the difference score correlated (see Table 2), which can be explained by younger participants on average in the BB condition. Pretest, posttest, and difference scores correlated as expected.

**TABLE 2:** Pearson’s correlations for condition, gender, age, pretest and posttest balance beam scores, and the difference between pretest and posttest.

	1	2	3	4	5	6
(1) Condition	−					
(2) Gender	0.06	−				
(3) Age	−0.18*	–0.10	−			
(4) Pretest	0.06	–0.04	0.15	−		
(5) Posttest	0.33**	0.09	–0.05	0.56**	−	
(6) Difference score	0.28**	0.13	−0.20*	−0.49**	0.45**	−

*p < 0.05, **p < 0.01.

To further explore age and gender effects, a set of regression analyses were conducted. Given the sample size, using age and gender as covariates in the LTA was not feasible. The regressions had condition, age, gender, and all their interactions as independent variables that were related to total correct on pretest and posttest and the difference score as dependent variables. Both forward and backward regression were conducted, and the results were the same. Regarding the total correct on pretest, there was an interaction effect of age and condition, β = 0.17, *t*(144) = 2.02, *p* = 0.045. Follow-up analyses showed that total correct on pretest and age were correlated in the BB condition, *r*(101) = 0.23, *p* = 0.020, but not in the control condition, *r*(43) = −0.020, *p* = 0.898. This indicated that in the BB condition, which included more and younger children on average, age and balance beam understanding were positively related.

With reference to the total correct on posttest, only condition showed a significant relation, β = 0.39, *t*(144) = 5.08, *p* < 0.001. Total correct on posttest was larger in the BB compared to the control condition; see Table 1.

Concerning the difference score, the effect of condition, β = 0.68, *t*(143) = 3.11, *p* = 0.002, and the interaction of condition with age were significant, β = −0.44, *t*(143) = 2.01, *p* = 0.047. Difference scores were larger in the BB compared to the control condition; see Table 1. Follow-up analyses showed that the difference score and age were correlated in the BB condition, *r*(101) = −0.21, *p* = 0.031, but not in the control condition, *r*(43) = −0.020, *p* = 0.902. This indicated that in the condition that showed learning, the younger the child, the more he or she learned, on average.

### Learning Gains

To verify that learning occurred, pretest and posttest scores were analyzed while taking condition into account in a mixed-design ANOVA. Condition (control vs. BB) was the between-subjects factor, and time (pretest vs. posttest) the within-subjects factor. First, age was added as a covariate to control for possible age effects. The interaction of age with time and the three-way interaction of age, condition, and time were not significant, *F* < 1, *p* > 0.350 in all cases. Therefore, the repeated-measures ANOVA was conducted without age. The main effect of time was not significant, *F*(1,155) = 0.97, *p* = 0.327, $\eta_{\text{p}}^{2}$ = 0.006, but the interaction of time and condition was significant, *F*(1,155) = 13.36, *p* < 0.001, $\eta_{\text{p}}^{2}$ = 0.079. This interaction effect can be explained by higher posttest scores in the BB condition, while pretest scores did not significantly differ from the control condition; see Table 1. To further explain this result, pretest and posttest scores were compared, and posttest scores were higher than pretest scores in the BB condition, *t*(112) = 4.72, *p* < 0.001, *d* = 0.38, but not in the control condition, *t*(43) = 1.35, *p* = 0.184, *d* = 0.22. The effect in the BB condition was small (Cohen, 1992).

### Latent Transition Analysis

Before analyzing change in strategy use, the number of classes was determined. This was determined based on both pretest and posttest performance. LTA was conducted with condition as a covariate of transitions from pretest to posttest because condition affected learning gain. To verify that condition did not affect class assignment on pretest, LTA was conducted with condition as a covariate of initial probabilities as well; condition did not affect these probabilities. In other words, for any class at pretest, children were as likely to be assigned to it in the BB as in the control condition. Thus, LTA was conducted with condition as a covariate of transitions only. To determine the number of classes, the fit of models with two to seven classes was investigated using random initialization of the expectation-maximization algorithm, and when the optimal number of classes was found, a deterministic initialization was used (Bartolucci et al., 2017). In a first analysis, two classes appeared to be a result of outliers, as these classes were on either end of the scale (very low accuracy and very high accuracy). Furthermore, these classes were barely used (below 2%). Therefore, these four outliers (all in the BB condition) were removed, and LTA was conducted with a sample size of 153. The Bayesian information criterion (BIC), which is a good indicator of fit in models with latent variables (Nyland et al., 2007), supported a five-class model (i.e., the lowest BIC); see Table 3. Using different starting values did not make a difference; the five-class solution had the lowest BIC. An entropy of 0.89 for this solution indicated that separation between the latent classes was good, as it approached 1 (Celeux and Soromenho, 1996).

**TABLE 3:** Fit of the models with two to seven classes.

Number of classes	Number of free parameters	Log-likelihood	AIC	BIC
2	55	−3,787.38	7,684.75	7,851.43
3	89	−3,499.31	7,176.63	7,446.33
4	127	−3,478.22	7,210.45	7,595.31
5	169	−3,195.29	6,728.57	7,240.71
6	215	−3,153.34	6,737.80	7,389.34
7	265	−3,071.76	6,673.51	7,476.58

**AIC stands for Akaike information criterion, and BIC stands for Bayesian information criterion*.*

#### The Classes: Modeling Strategy Use

**FIGURE 3:**
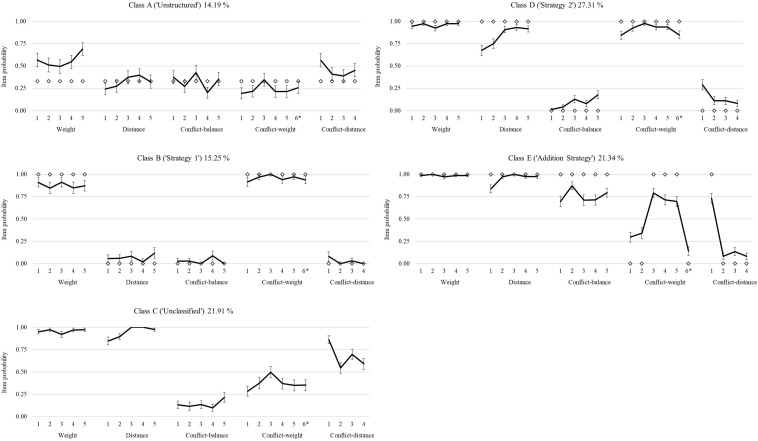
Item probabilities for the six classes of the latent transition analysis (LTA), with the corresponding strategy and percentage of usage at pretest, and corresponding 90% confidence intervals. Items were distance, conflict-weight, conflict-distance, and conflict-balance; each had five items. *Note that item 5 of conflict-distance was digitized incorrectly and was a conflict-weight item.

Response patterns (based on the item probabilities) of the five classes were investigated in order to characterize the classes; see Figure 3. Figure 3 depicts how children in the different classes scored on all items of the balance beam test. Class D was used most often. Class E had the highest overall accuracy on the balance beam test; see Table 4.

**TABLE 4:** Classes, their names, usage, and overall correct at pretest.

Class	Class name	Usage at pretest	Overall correct
A	Unstructured	14.19%	37.22%
B	Strategy 1	15.25%	42.87%
C	Unclassified	21.91%	60.51%
D	Strategy 2	27.31%	61.88%
E	Addition strategy	21.34%	69.92%

Class A did not match any of the proposed strategies, and it was called “Unstructured.” The pattern did not reveal consistency in the responses. Accuracy on all items was around chance (0.33 item probability). The only exception was weight, where performance was slightly better. Class A, however, does not match Strategy 1 (use weight only in predicting which side goes down), because accuracy on conflict-weight items is around chance. This is interesting to note, because such patterns would not be visible when excluding the weight-only items (cf. Boom et al., 2001). Class A was used 14.19% of the time on pretest.

Class B had a good fit with Strategy 1, using weight only and ignoring distance, and was, therefore, called “Strategy 1.” This can be seen by the high accuracy on weight and conflict-weight items, while accuracy on other items is around zero. This large difference in accuracy between weight and non-weight items is characteristic of Strategy 1. Class B was used 15.25% of the time on pretest.

Class C did not match any of the strategies proposed by Siegler (1976) and was called “Unclassified.” Class C could be indicative of Strategy 3 to some extent: using both weight and distance but in an inconsistent manner. Class C showed a large resemblance to class 3 found by Boom et al. (2001). The item probabilities were large for weight and distance items, indicating that most participants in this class were able to solve these problems. The confidence intervals were larger for the conflict items than for non-conflict items, indicating that there was a larger variability of responses on conflict items. Conflict-distance item 1 had a relatively high accuracy compared to the other conflict-distance items, which might be explained by the relatively high torque of this item; see Appendix A for item details. Class C was used 21.91% of the time on pretest.

Class D had a good fit with Strategy 2 (i.e., use weight and, when weights are equal, use distance) and was called “Strategy 2.” The item probabilities indicated high accuracy on weight and conflict-weight items. Accuracy on distance items was high and close to 100% for three items. The overall pattern of class D showed a good match with class 2 from Boom et al. (2001). Class D was used 27.31% of the time on pretest.

Class E had a reasonable fit with the Addition Strategy. When using the Addition Strategy, weight and distance are added on each side, and subsequently, these sums are compared. All conflict-balance items; conflict-weight items 3, 4, and 5; and conflict-distance item 1 should be answered correctly when using the Addition Strategy. Accuracy on these items in class E was around 75% correct. Boom et al. (2001) also found a class matching the Addition Strategy: class 4. Class E was used 21.31% of the time on pretest.

To summarize, classes B and D were shown to be a good match to Strategies 1 and 2 proposed by Siegler (1976). Class E matched the Addition Strategy (Wilkening and Anderson, 1982). Class C did not match any strategy, but it is in line with previous studies that found such classes (e.g., Boom et al., 2001). Class A showed an unstructured pattern and did not match any previously detected strategy.

Each child was assigned a probability to belong to the classes. There were 112 children who were assigned to a single class (over 99% assignment). Additionally, 35 children had a 75% or higher assignment to a single class. Only six children had a lower than 75% probability to belong to one class, but the lowest probability for these children was over 50%. At posttest, these numbers were comparable: 113 children over 99%, 32 children over 75%, 7 children between 50% and 75%, and one below 50%. The one child with class assignment below 50% on posttest was assigned to class C (48%) and class E (48%), whose response patterns appeared to overlap.

#### The Transitions: Modeling Change

Change in strategy use was examined in more detail by investigating the transition from one class at pretest to another at posttest. Transition probabilities indicate the probability of moving from a specific class to another class; see Table 5 for the transition probabilities for the control and BB conditions combined. Most class assignments did not change; see the diagonal. For example, 76% of class E (Addition Strategy) remained class E at posttest. Although there were some declines, mainly from Strategy 2 to Unclassified (20%), most changes in class indicated use of a better strategy, such as from Strategy 1 to Unclassified and from Unclassified to Addition Strategy. How these effects could be explained by condition was investigated next.

**TABLE 5:** Transition probabilities for classes from pretest to posttest.

		Posttest class				
		Unstructured	Strategy 1	Unclassified	Strategy 2	Addition strategy
Pretest class	Unstructured	0.49	0.12	0.22	0.11	0.06
	Strategy 1	0.09	0.25	0.28	0.25	0.14
	Unclassified	0.06	0	0.64	0	0.30
	Strategy 2	0.15	0.02	0.20	0.60	0.03
	Addition strategy	0.10	0	0.09	0.06	0.76

**FIGURE 4:**
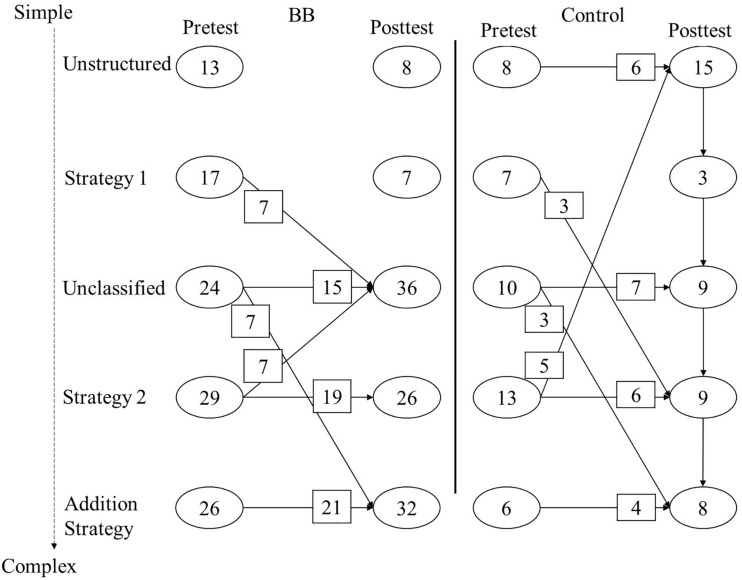
Number of children per class for pretest and posttest with the transitions for the control and balance beam (BB) conditions. Transitions smaller than 5% of the sample were omitted from the figure for parsimony; see Appendix B for all transitions.

The number of children who transitioned was calculated for each condition. To do so, children were assigned to a single class, based on the largest probability to belong to a class. Similarly, transition probabilities per condition were requested and transformed into the number of children who transitioned. In both conditions, most children did not change their strategy; see Figure 4. In the BB condition, there were more transitions that indicated advancement in strategy compared to the control condition. In addition, the advancements indicated larger increases in accuracy on average, and the declines indicated smaller decreases in accuracy compared to the control condition. All transitions indicating improvement were present in the BB condition, except for the transition from Unclassified to Strategy 2. This cannot be seen in Figure 4, as small transitions were omitted for parsimony, but see Appendix B for all transitions per condition. To summarize, 65 children kept using the same strategy, 16 children declined, and 28 children improved in the BB condition.

## Discussion

The aim of the present study was to examine conceptual change as a result of completing an inquiry-based lesson about balance beams compared to a control condition that entailed completing an inquiry-based lesson about gears. In particular, the changes in strategy use between pretest and posttest when solving balance beam problems were examined. The results showed that classes could be detected that generally matched the strategies proposed by Siegler (1976) and the classes found by Boom et al. (2001). In addition, children in the BB condition showed advances in understanding the law of moments, and no learning occurred in the control condition on average. Additional insights were obtained by detecting transitions between classes from pretest to posttest with LTA. LTA showed how condition affected learning: transitions to complex and accurate strategies occurred more often in the BB condition. Most improvements in the BB condition could be attributed to the adoption of the Addition Strategy (Wilkening and Anderson, 1982) and the “Unclassified” class, which showed some resemblance to Strategy 3. Together, these results showed that a single session of inquiry-based learning about the balance beam induced conceptual change in about 26% of the children, that conceptual change can be measured as advancement (or for some, a decline) in strategy use, and that this can be modeled using latent models.

The classes that were found corresponded reasonably well with the strategies proposed by Siegler (1976) and the classes found by Boom et al. (2001). There were two classes that approximately matched the strategies proposed by Siegler (1976): classes B and D, which matched Strategies 1 and 2. Four out of six classes identified by Boom et al. (2001) were found in the present study. These were classes revealing Strategies 1 and 2 and the Addition Strategy, and an Unclassified class. Furthermore, these classes were used as often in the present study as by the 11.4-year olds in the study by Boom et al. (2001), with only the Unclassified class being used more often in the present study. Most children used Strategy 2 (27%), the Addition Strategy (21%), or the Unclassified strategy (22%). Taken together, the results show that Strategy 2 and the Addition Strategy are common strategies that can be identified in 9- to 11-year-old children.

Class A deviated most from previous studies. Class A (“Unstructured”) showed a pattern that did not reveal consistency in the responses. The response pattern appeared to suggest guessing, which might be explained by randomly selecting an answer without carefully thinking. This could mean that children in this class did not know the answer on any of the items. Previous studies show at least understanding of weight as a factor in balancing beams (e.g., Boom et al., 2001). Class A did show accuracy above chance for weight. Therefore, children in this class did show some understanding, but the response pattern did not fit any strategy. An explanation of the low accuracy might be the young age of the present sample in combination with no previous experience with the law of moments.

The Unclassified class did not show a good match to Siegler (1976) strategies, but the present results support some overlap with Strategy 3: using both weight and distance but in an inconsistent manner. One result supporting the overlap with Strategy 3 is that items with weight only and distance only were answered correctly in this class, while there was large variation in the conflict items, where weight and distance have to be combined to answer the items correctly. Another reason is that there were no transitions to Strategy 2 (class D), where weight is the dominant variable in predicting which side goes down and distance is only used when weights are equal, but there were transitions to the Addition Strategy (class E). It can therefore be speculated that children using the Unclassified class had more advanced understanding than those using Strategy 2, even though their overall accuracy was slightly lower.

No class was found matching Strategy 4: perfect accuracy (Siegler, 1976). This might be explained by the age of the children in the present study: 10 years and 9 months on average. In another study, 11.4-year olds barely used Strategy 4: around 2% (Boom et al., 2001). It was expected that improved accuracy on the balance beam test as a result of the inquiry-based lesson about balance beams would also result in some children using Strategy 4. This did not happen, which suggested that acquisition of the most complex strategy in this age group is unlikely after a single inquiry-based lesson.

The present results showed that on average, children performed better on the balance beam test after the inquiry-based lesson about the balance beam than before, while those in the control condition did not improve. This means that a single session of inquiry-based learning could promote conceptual change, measured as a change in strategy use. This result can be explained by the effectiveness of inquiry-based learning (Lazonder and Harmsen, 2016). Others have also found beneficial effects of inquiry-learning (Huang et al., 2017) or problem-based learning (Loyens et al., 2015) on conceptual change. In the present study, the inquiry-based lesson consisted of elements that addressed activation of prior knowledge (hypothesis generation), critical analysis of findings (data interpretation), and deep comprehension (evidence evaluation). These elements were also found to be effective in problem-based learning (Loyens et al., 2015). The present inquiry-based lesson included additional supportive features: explication of predictions, self-generation of evidence, perceiving evidence, and verifying or falsifying understanding. These features target difficulties in conceptual change that were identified in primary school children (Vosniadou, 2002). The present study is one of the first to show that inquiry-based learning in primary school indeed fosters conceptual change.

A main result of this study was that improvement on the balance beam test could be attributed to specific transitions from one class to another, indicating that a different strategy was used after the inquiry-based lesson in the BB condition as compared to before. About 26% of the children in the BB condition advanced in strategy use. Most of these children who improved started with applying weight only (Strategy 1) and learned to use both weight and distance when weight was equal on both sides of the balance beam (Unclassified) or went from this Unclassified strategy to the Addition Strategy. While one improvement indicated an advancement of one step (Unclassified to Addition Strategy), there were numerous instances of improvements of more than one step, including from Strategy 1 to Unclassified, where Strategy 2 seems to be skipped. Note that although this is speculative, it showed that conceptual change does not have to be slow (Vosniadou, 2002). A different reason for change might be a different conceptualization of the problem and not a different conceptualization of the law of moments, which might be addressed in future studies. Nevertheless, this finding highlighted the advantage of using latent analyses, because overall improvement in the total score could be attributed to specific advancements in strategy use. In addition, the control condition did show transitions as well, although the total score did not change. It appeared that some children declined and some improved in strategy use. There might be multiple explanations for this finding. One explanation would be that children have access to multiple strategies at one time [see the overlapping waves model (Siegler, 2000)] and might switch to some extent from pretest to posttest. Another explanation is that responses to the problems are unstable because children do not have a coherent explanatory framework yet (Vosniadou, 2002).

Given that conceptual change occurred, it can be assumed that children experienced disequilibrium during inquiry-based learning. Disequilibrium is a moment in which the current explanatory framework is not adequate and children are open to new explanations (Piaget, 1978). After this moment, children can advance, or children can decline. The present results found both. In the case of a decline, new strategies entailed a less correct approach to the problems. This result resembled the results of Huang et al. (2017), who found that correct propositions were adopted after inquiry-based learning but not assembled together correctly. In the present study, children did seem to correctly identify the need for a new strategy, but they did not yet create a coherent explanatory framework that could be used to solve the problems.

A final result regarding the transitions was that about 60% of the children in the BB condition did not move to another class and, thus, did not change strategy use. This is in line with the small-sized effect (Cohen, 1992) of the inquiry-based lesson. The effect, however, is close to the average found in educational interventions (Hattie, 2012). It is likely that the children who did not move to another class did not experience disequilibrium (Piaget, 1978) and/or were not able to notice other potential explanatory variables (Siegler and Chen, 1998). This might be due to, for example, children’s scientific reasoning skills or the learning materials. It has been shown that the process of inquiry-based learning is performed better when children have strong scientific reasoning skills (Stender et al., 2018), but other factors that support scientific reasoning could affect the inquiry process as well, such as self-control or verbal skills (Van der Graaf et al., 2018), or Theory of Mind (Koerber and Osterhaus, 2019). Self-control and verbal skills might help to perform the most important process according Siegler and Chen (1998)—noticing potential explanatory variables—because it enables children to stop and think (self-control) and to access perceptual experiences via verbalization (verbal skills). On the other hand, the learning materials were effective overall but not for everyone. To improve learning for each child, the lesson might be repeated (Anderson, 1982), or additional support could be built in, for example, to reduce working memory load (Sweller et al., 2011), which could also lead to the acquisition of Strategy 4 in some children.

Exploratory analyses with age and gender were conducted. Gender did not relate to the pretest, posttest, or difference score (posttest minus pretest), which was in line with gender being not significant as a covariate in an LCA on the balance beam test (Boom and Ter Laak, 2007). The present results showed that gender also did not relate to a difference score. Age correlated positively with pretest scores and negatively with the difference score but only in the BB condition, in which children were slightly younger on average than in the control condition. Thus, in the younger group of children, young children appear to be less accurate in the balance beam test. This finding, again, is in line with previous studies on balance beam understanding (Boom et al., 2001; Boom and Ter Laak, 2007). What the present results add is that when learning occurs (i.e., in the BB condition), younger children tend to learn more. This result can best be interpreted with the relation between age and pretest scores in mind, which means that children with low pretest scores have large difference scores. Inquiry-based learning has previously been shown to result in larger learning gains for low-prior-knowledge children (e.g., Wang et al., 2010). As it is yet unknown what the effect of age on transitions from pretest to posttest would be after an inquiry-based lesson about the balance beam, it would be interesting to analyze latent classes, class assignment, and transitions, while using age as a covariate of class assignment, as Boom and Ter Laak (2007) did, and of transitions as well.

Some limitations apply to the present study. One limitation was the number of participants. As their response patterns were used to identify classes and this could change from pretest to posttest, more participants would be preferable to be better able to identify additional classes, especially classes that might have existed at pretest only or that emerged at posttest. There is no definite rule for sample size in LCA, because it also depends on circumstances, but sample size should be at least 70 participants (Hickendorff et al., 2018). The current number of participants is justified by classes consisting of at least 20 children, and classes were previously identified with larger sample sizes (Boom et al., 2001; Boom and Ter Laak, 2007). A better number of participants, especially when using covariates, would be around 500 participants (e.g., Boom and Ter Laak, 2007). Another limitation is that the actions of the learners were not recorded. It might be that children who designed the experiments as instructed learned more. A third limitation is that individual differences beyond age and gender were not taken into account. It is likely that the inquiry-based lesson required children to use their working memory (e.g., Kwon and Lawson, 2000) because inquiry-based learning activities involve, amongst others, use of prior knowledge and tracking manipulations of variables and experimental outcomes. In the present study, working memory demands were reduced by allowing hypotheses, results, and conclusions to be written down, allowing the revisiting of previously presented information, and more. Instruction in the digital learning environment was presented using text, which means that reading levels might have affected the results, possibly as a moderator (Graesser et al., 2005). However, reading levels were not assessed in the present study.

A strength of the present study was that transitions from pretest to posttest in strategy use when solving balance beam problems were uncovered. These transitions indicate that conceptual chance occurred. While more research is needed, the present results showed that it is feasible to assess conceptual change with the balance beam test after a short inquiry-based lesson about balance beams.

In the future, a feature might be added to prevent overloading working memory, namely, note-taking, especially when comparing the products (weight multiplied by distance) of the two sides of the balance beam. Note-taking (vs. not note-taking) might be especially advantageous for transfer to problem solving tests (Peper and Mayer, 1986). In addition, reviewing notes that were written down during the experiment positively relates to science understanding (Klein, 2000). An extended intervention could also be analyzed using LTA, which can show whether repeated exposure to the correct scientific theory induces additional advancement. LTA can reveal transitions between classes. LTA can also reveal which newly acquired strategy at posttest is most likely to be disregarded at a later time, if there is a decline in accuracy, as can happen in delayed posttests. It might be expected that a strategy corresponding to Strategy 3 or 4, where weight and distance are combined, is more difficult to learn and retain than Strategy 2, and therefore, it might be used at posttest but not anymore at delayed posttest. A final suggestion would be to investigate individual differences in strategy use and change. Covariates can be used in LTA to investigate this, for example, understanding of a correct experimental strategy positively related to knowledge about floating and sinking and to the probability to change to a more proficient knowledge profile (Edelsbrunner et al., 2018).

The results suggested that for classroom science education, it is important to take into account differences between and within individuals at a specific time and over time (Hickendorff et al., 2018). The classes showed the existence of multiple strategies in a group of children (Jansen and van der Maas, 1997). Some classes suggested a mixture of strategies being present at one time (Siegler, 2000). Some children improved their strategy use when predicting which side of a balance beam goes down. Therefore, a suggestion would be to present digital inquiry-based learning activities in which the learning process is recorded to get insight into explanatory frameworks in use (cf. diSessa, 2002), to get insight into optimal learning moments (disequilibrium; Piaget, 1978), and to provide teachers with potential learning paths for the heterogeneous group of children being taught. Another suggestion is to focus on how to combine weight and distance to correctly calculate momentum. In the present study, most children in the BB condition understood that both variables matter at posttest but did not know how to combine them correctly.

To conclude, strategy use when solving problems with the balance beam could be identified and the classification reasonably matched with the strategies proposed by Siegler (1976). The strategy that was used changed after an inquiry-based lesson about balance beams for about 40% of the children, indicating that conceptual change occurred. It is, therefore, feasible to assess conceptual change with the balance beam test after inquiry-based learning in primary school.

## Data Availability Statement

The datasets generated for this study are available on request to the corresponding author.

## Ethics Statement

The studies involving human participants were reviewed and approved by BMS Ethics Committee, University of Twente. Written informed consent to participate in this study was provided by the participants’ legal guardian/next of kin.

## Author Contributions

The author confirms being the sole contributor of this work and has approved it for publication.

## Conflict of Interest

The authors declare that the research was conducted in the absence of any commercial or financial relationships that could be construed as a potential conflict of interest.

## Footnotes

1 Three items deviated from the study by Boom et al. (2001), due to incorrect digitization. Two items did not change in type, but one item did, namely, item 25 about conflict-distance. It was therefore a conflict-weight item; see Appendix A for all item details. Two items were identical, namely, items 16 and 25. Since accuracy was highly similar and an additional item provided more information for the analysis, item 25 was not removed.

2 https://phet.colorado.edu

## Appendix A

**TABLE A1:** Item characteristics: number, type, weight, and distance on the left and right sides of the balance beam, torque, and which side goes down.

		Item configuration						
		Left		Right				
Item number	Item type	*D*	*W*	*D*	*W*	Torque	Side down	Addition strategy
1	W	2	1	2	2	2	Right	Correct
2	W	3	3	3	4	3	Right	Correct
3	W	1	2	1	4	2	Right	Correct
4	W	2	3	2	2	−2	Left	Correct
5	W	4	3	4	1	−8	Left	Correct
6	D	2	1	1	1	−1	Left	Correct
7	D	3	2	2	2	−2	Left	Correct
8	D	4	3	2	3	−6	Left	Correct
9	D	3	4	4	4	4	Right	Correct
10	D	2	3	3	3	3	Right	Correct
11	CB	1	4	4	1	0	Balance	Correct
12	CB	3	1	1	3	0	Balance	Correct
13	CB	3	2	2	3	0	Balance	Correct
14	CB	3	4	4	3	0	Balance	Correct
15	CB	4	2	2	4	0	Balance	Correct
16	CW	4	2	3	3	1	Right	Incorrect
17	CW	2	3	4	1	−2	Left	Incorrect
18	CW	3	1	2	3	3	Right	Correct
19	CW	1	4	3	1	−1	Left	Correct
20	CW	3	2	2	4	2	Right	Correct
21	CD	1	4	3	3	5	Right	Correct
22	CD	2	2	1	3	−1	Left	Incorrect
23	CD	3	3	2	4	−1	Left	Incorrect
24	CD	1	4	2	3	2	Right	Incorrect
25	CW*	4	2	3	3	1	Right	Incorrect

**W, weight; D, distance; C, conflict. *This CW item should have been a CD item but was digitized incorrectly. The number of weights is presented, and each weight was 5 kg*.*

## Appendix B

**TABLE B1:** Number of children in the control condition who transitioned from pretest to posttest.

		Posttest class				
		Unstructured	Strategy 1	Unclassified	Strategy 2	Addition strategy
Pretest class	Unstructured	6	2	0	0	0
	Strategy 1	2	1	0	3	1
	Unclassified	0	0	7	0	3
	Strategy 2	5	0	2	6	0
	Addition strategy	2	0	0	0	4

**TABLE B2:** Number of children in the balance beam (BB) condition that transitioned from pretest to posttest.

		Posttest class				
		Unstructured	Strategy 1	Unclassified	Strategy 2	Addition strategy
Pretest class	Unstructured	5	1	4	2	1
	Strategy 1	0	5	7	3	2
	Unclassified	2	0	15	0	7
	Strategy 2	1	1	7	19	1
	Addition strategy	0	0	3	2	21
